# Old innovations and shifted paradigms in cellular neuroscience

**DOI:** 10.3389/fncel.2024.1460219

**Published:** 2024-08-21

**Authors:** Riccardo Fesce

**Affiliations:** Department of Biomedical Sciences, Humanitas University Medical School, Pieve Emanuele, Italy

**Keywords:** neurotransmitter release, electrophysiology, patch clamp, freeze-fracture, noise analysis, fluorescent dyes, kiss and run

## Abstract

Once upon a time the statistics of quantal release were fashionable: “*n*” available vesicles (fusion sites), each with probability “*p*” of releasing a quantum. The story was not so simple, a nice paradigm to be abandoned. Biophysicists, experimenting with “black films,” explained the astonishing rapidity of spike-induced release: calcium can trigger the fusion of lipidic vesicles with a lipid bilayer, by masking the negative charges of the membranes. The idea passed away, buried by the discovery of NSF, SNAPs, SNARE proteins and synaptotagmin, Munc, RIM, complexin. Electrophysiology used to be a field for few adepts. Then came patch clamp, and multielectrode arrays and everybody became electrophysiologists. Now, optogenetics have blossomed, and the whole field has changed again. Nice surprise for me, when Alvarez de Toledo demonstrated that release of transmitters could occur through the transient opening of a pore between the vesicle and the plasma-membrane, no collapse of the vesicle in the membrane needed: my mentor Bruno Ceccarelli had cherished this idea (“kiss and run”) and tried to prove it for 20 years. The most impressive developments have probably regarded IT, computers and all their applications; machine learning, AI, and the truly spectacular innovations in brain imaging, especially functional ones, have transformed cognitive neurosciences into a new extraordinarily prolific field, and certainly let us imagine that we may finally understand what is going on in our brains. Cellular neuroscience, on the other hand, though the large public has been much less aware of the incredible amount of information the scientific community has acquired on the cellular aspects of neuronal function, may indeed help us to eventually understand the mechanistic detail of how the brain work. But this is no more in the past, this is the future.

## 1 Introduction

The field of Cellular Neuroscience has experienced an intense evolution for many years now, and novel approaches and techniques emerge every month. Having retired last year, the idea of “Paradigm Shifts and Innovations in Cellular Neuroscience” provoked me to look back to the old innovations I witnessed and the paradigm that shifted during my scientific career.

## 2 Vesicle fusion and the “fusion machine”

During my stage at the Rockefeller University in New York I joined a great group of biophysicists, headed by Alex Mauro. They had been working for quite some time on the biophysics of biological membranes (Mauro and Finkelstein, 1958) and generating artificial membrane models (the so-called “black films”). They observed that phospholipid vesicles could be induced to fuse with such films by raising calcium concentration, so that their content would be released on the other side of the membrane (Zimmerberg et al., 1980) and components of the vesicle membrane would be incorporated into the planar membrane itself (Cohen et al., 1980). It had long been known that release of neurotransmitter evoked by the presynaptic action potential depends on the presence of calcium ions (Fatt and Katz, 1952); also, the seminal work of Del Castillo and Katz (1954) had clarified that transmitter release occurs in “packets” of relatively constant size (“quanta”); when the fine structure of the neuromuscular junction was revealed by electron microscopy, the idea had come about that the fusion of a synaptic vesicle could be the structural equivalent of the recording of a “quantal” event at the postsynaptic membrane. Thus, the action of calcium in favoring phospholipid vesicle fusion with planar membranes was taken to suggest that calcium ions favor phospholipid membrane fusions, possibly by shielding the fixed – predominantly negative – charges of the phospholipids.

For several years, a silent debate went on between biochemists and physicists, about synaptic vesicle fusion: neurotransmitter release occurs in less than a ms after the action potential invades the nerve terminal, so biophysicists would not even consider the possibility that complex biochemical events be interposed between the nerve terminal depolarization and the fusion of vesicles; the instantaneous masking action by calcium ions on the fixed charges of the facing membranes seemed more likely. On the other hand, biochemists were isolating proteins specifically expressed at synapses and presumably involved in quantal release of transmitter (e.g., Südhof and Jahn, 1991); also, biochemical processes clearly influenced quantal release, and clostridial toxins (tetanus and botulinum) were known to impair neuromuscular transmission, through an unclear mechanism (see, e.g., Burgen et al., 1949; Brooks, 1954; Harris and Miledi, 1971) that clearly impaired a step between calcium entry and transmitter release (Dreyer and Schmitt, 1983; Bevan and Wendon, 1984).

**FIGURE 1:**
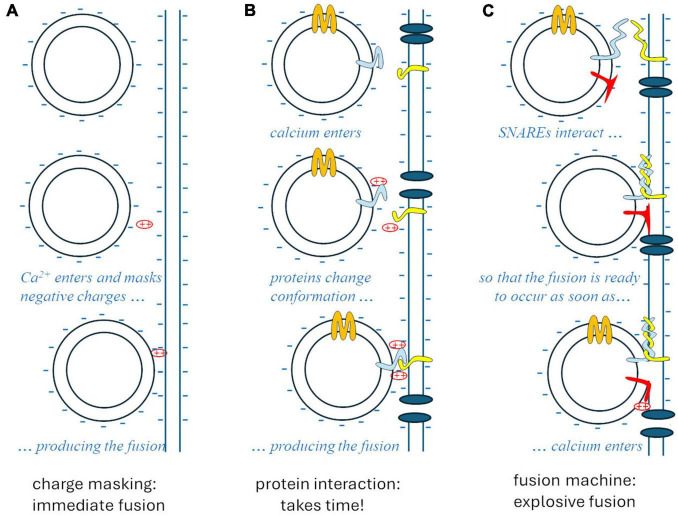
The theories of synaptic vesicle fusion. **(A)** The “biophysical” hypothesis, Ca^2++^ masking the negative charges of the membrane phospholipids, would account for the speed (<1 ms) but not for specificity and biochemical regulation. **(B)** The “biochemical” hypothesis would account for specificity and regulations but would take too long after the entry of Ca^2+^. **(C)** The SNARE model accounts for specificity, regulation, and the explosive speed, due to the high free-energy intermediate generated by the “fusion machine.”

The whole story was clarified by Rothman’s studies on vesicular traffic in the cell (Rothman, 1994; Söllner et al., 1994), disorganized by N-ethylmaleimide. This substance interferes with an ATPase, referred to as N-ethylmaleimide sensitive factor (NSF), which is needed for the specific exo-endocytosis of vesicles at the membranes they are supposed to fuse with. Such recognition relies on complementary proteins that are expressed in intracellular vesicle membranes and in target membranes: these proteins bind cytoplasmic interactors of NSF (Soluble NSF-Attachment Protein, SNAPs), so they are called SNAP receptors, respectively vesicular (v-SNARE) and target (t-SNARE). The interaction between the tails of v- and t-SNARE proteins acts like a zip that pushes the two membranes against each other, generating a high free-energy conformation that strongly favors the fusion of the two membranes. At synapses, such fusion is prevented by a “brake” protein – presumably synaptotagmin – until a simple conformational change of the latter, produced by the interaction with calcium ions, allows the strongly thermodynamically favored fusion to occur, explosively (Figure 1).

In the meanwhile, Cesare Montecucco and his coworkers were studying clostridial toxins (Montecucco and Schiavo, 1993) and observed a curious property of theirs: they acted as zinc proteases; it turned out that tetanus toxin and botulinum toxins B and D acted on VAMP-synaptobrevin, an integral membrane protein of synaptic vesicles, which Rothman had independently identified as a synaptic v-SNARE; on the other hand, botulinum toxins A and E cleaved SNAP-25, and toxin C cleaved syntaxin, having both proteins been identified by Rothman as presynaptic membrane t-SNAREs (Montecucco and Schiavo, 1995).

This new paradigm finally reconciled the two opposed views: a complex preliminary biochemical process is actually needed for the “fusion machine” to mature around the v- and t-SNARE complex, involving other proteins, among which the N-type calcium channel; once the complete multiprotein apparatus is formed, however, the fusion and the subsequent synchronous release of neurotransmitter occurs instantly in response to the depolarization of the nerve terminal, thanks to a high free-energy conformation produced by the strong interaction between complementary proteins that push the vesicle and plasmatic membrane against each other.

The idea that the synaptic vesicle protein synaptotagmin (Syt1) could be the one triggering fusion in a calcium-dependent way was put forward quite early (Perin et al., 1990), because of its capability to interact with phospholipids in a Ca^2+^-dependent way, although – or properly because – it has low affinity for Ca^2+^ (the local concentration of the ion in the microdomain close to the active rises high, when Ca^2+^ enters through the N-type channels opened by the action potential, and the conformational changes must occur quite quickly, presumably in less than 100 μs). Still, it took some 20 years to Nobel laureate Südhof and his group, and other laboratories involved in this research, to put the puzzle together and clarify the quite complex machinery involved in the process. Synaptotagmin was confirmed to be the key molecule, through its interaction with membrane phospholipids, especially for the synchronous secretion mediated by the action potential. However, more and more proteins were gradually shown to be involved. For example, proper conformation of syntaxin, through interaction with Munc18-1and Munc13, was shown to be needed to prime the docked vesicles for fusion; complexin was shown to be involved in the “clamping” action of Syt-1 on the SNARE complex; the G-protein Rab3 was shown to guide the vesicle to the active zone by interacting with RIM proteins, which also recruit Ca^2+^-channels to the active zone, mediate synaptic plasticity and activate Munc13 proteins. In summary, a quite complex network of mutually interacting proteins (Südhof, 2012), something decidedly different from a simple electrostatic interaction between calcium ions and the negative charges of membrane phospholipids…

## 3 Quantal release and the “binomial” paradigm

Entering the field of Cellular Neuroscience as an electrophysiology scholar, I was fascinated by the work of Sir Bernard Katz and his colleagues on quantal release of neurotransmitter. At that time, our lab, directed by Bruno Ceccarelli, was mostly committed to correlating electron microscopy data with electrophysiological recordings, with the purpose of verifying what was referred to as “the vesicle hypothesis of quantal release” of neurotransmitters.

Personally, I was intrigued by the idea, quite fashionable then, that the quantal content of the end-plate potential (EPP) in successive trials could be described by binomial statistics, where the binomial parameters *n* and *p* would refer to, respectively, the number of available vesicles (or fusion sites) and the probability for the vesicles to fuse with the presynaptic membrane and release the neurotransmitter they contained. This interpretation of the binomial statistics of evoked quantal release became very popular after several processes of facilitation (a fast and a slow component of facilitation, augmentation, and post-tetanic potentiation; Magleby, 1979) and depression of quantal release were described in paired-pulse experiments, by comparing the sizes of the responses to two stimuli given in rapid sequence, or by examining the time course with which a synapse would return to the basal secretory activity following repetitive stimulation. Everything seemed consistent with the idea that stimulation increased the probability for vesicles to fuse (increasing *p* due to residual calcium ions and/or to phosphorylation of various substrates) but this effect could be counteracted by depletion of the available vesicles (decreased *n*, which may predominate on facilitation for intense quantal release).

However, some curious phenomena were apparent during repetitive stimulation: (i) in reduced calcium concentration (a situation that depresses quantal release) the EPP size would increase during the train, but binomial analysis would indicate an increase in *n* rather than release probability *p*; (ii) in normal calcium concentration, conversely, both *n* and *p –* not only *n* – would decrease during a high-frequency (50 Hz) train; (iii) the rate of occurrence of spontaneous events (miniaturized EPP, mEPP) between successive stimuli declined less than the quantal content of the EPPs (mEPP rate of occurrence even increased initially, at high stimulation frequency), suggesting that mechanisms regulating synchronous release (EPP size) and asynchronous release (mEPP rate) were somewhat different; (iv) finally, the *n-p* model would predict a strong correlation between successive responses in a train of stimuli: an EPP stochastically higher than the average should produce a momentary decrease in the number of synaptic vesicles (or sites) ready for release, *n*, and be followed by one or more responses lower than the average; but such a result was never reported. Did nobody think of this? more likely, negative results never get published, and several researchers probably wasted some time – as I did myself – in trying and detecting such kind of correlations. So, the variability of evoked quantal release did essentially follow binomial statistics, but the paradigm “*n* corresponds to the number of available vesicles (or fusion sites), while *p* represents the probability of release associated to each available vesicle (fusion site)” did not seem tenable.

**FIGURE 2:**
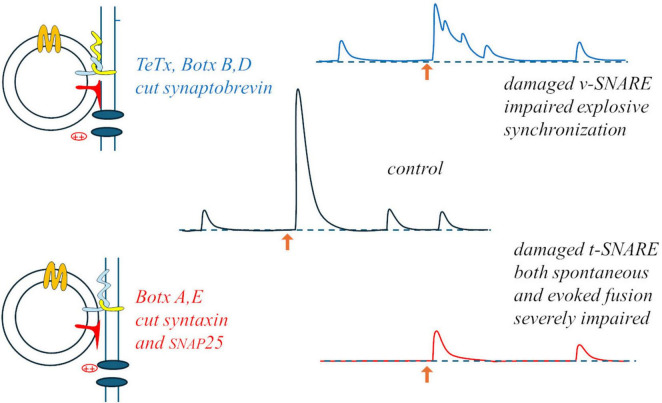
Differential effects of tetanus and botulinum toxins: damage to the v-SNARE may mostly impair the normally instantaneous and deterministic fusion upon Ca^2+^ entry **(upper part)**; the stochastic aspect may concern the formation of the fusion machine, strongly hampered by damaging the t-SNARES, and equally impairing both spontaneous and evoked release **(lower part)**.

Something interesting occurred when a neuromuscular preparation was subjected to “random-interval” repetitive stimulation: during a train of stimuli at 15 Hz average frequency (randomly variable intervals with average duration 67 ms), in 0.4 mM Ca^2+^ (20% of the normal Ringer’s concentration), average EPP size increased with a bi-exponential time-course (time-constants: 75 and 175 s). As expected from the results of paired-pulses experiments, the size of each EPP was significantly influenced by the duration of the preceding interval (but not by the size of the previous EPP): facilitation reached a maximum for 15 ms intervals (∼40% above the momentary average), waned by for 50 ms intervals, and EPP sizes fell to ∼40% below average for 200 ms intervals (Fesce, 1999). This indicated that during repetitive activation each impulse was able to produce a brief silencing (few ms) followed by momentary and transient – but relevant – increase in synaptic efficiency. A similar brief silencing, followed by transient activation, was observed in mEPP occurrences after each evoked response. These observations suggest that whatever can be released is released by the arrival of the action potential to the nerve terminal, in a deterministic rather than stochastic way, and that the stochastic aspects must be related to some steps that precede the evoked response, i.e., some steps involved in the formation of the “fusion machine” through the interaction among vesicular and target SNAREs, Munc proteins, synaptotagmin and complexin, in close proximity with the calcium channels. The specific action of clostridial toxins on the various synaptic SNAREs supports this view and may lead to revise the interpretation of the binomial statistics: botulinum toxin A (BoTx A), which cleaves syntaxin and SNAP25, produces a strong decrease in both spontaneous and evoked release, but the latter remains synchronous; conversely, tetanus toxin (TeTx), that cleaves synaptobrevin, produces a milder inhibition but totally desynchronizes evoked release, transforming it into bursts of mEPPs (Figure 2). Thus, it is likely that decimating the structures on the presynaptic membrane that can host a vesicle for fusion (BoTx A) does not interfere with the capability of the few remaining ones to produce synchronous, deterministic fusions; vice versa, damaging the vesicle capability to dock (TeTx) would make the fusion upon arrival of the action potential and Ca^2+^ entry a stochastic asynchronous event.

One may wonder what are the significance and the physiological role, if any, of the spontaneous occurrence of miniature events (minis). They could have a trophic role on the synapse, as suggested by the degeneration of the endplate (the postsynaptic membrane of the neuromuscular junction) when a muscle is denervated. However, such a role is presumably played by the continuous molecular leakage of neurotransmitter that does occur at synapses, aside from the spontaneous quantal release through synaptic vesicles that produces electrophysiologically recordable minis. Since the rate of occurrence of minis is strictly related to membrane depolarization (and Ca^2+^ entry, presumably) spontaneous release may be a remnant, in spike-dependant synapses, of the mechanism of quantal release at ribbon synapses, which do not produce synchronous release but rather modulate the frequency of miniature events. On the other hand, interfering with the proteins involved in the complex organization of the fusion machine produces differential effects on spontaneous and evoked release (e.g., Südhof, 2012), as it was also mentioned above for the effects of the various clostridial toxins. This suggest that the synapse may be able to regulate the intensity of spontaneous quantal release, the ratio between basal and evoked release, and thus the dynamic range of synaptic transmission. Such an effect may be relevant at central synapses where the quantal content of the postsynaptic potential is very low (corresponding to few minis) and even single minis, typically occurring at higher rate just after a synchronous release, may have some functional (electrical) effect.

A collateral aspect is that images of vesicle fusion on the presynaptic membrane are observed when the frequency of randomly occurring minis is artificially increased at the frog neuromuscular junctions: while fusions produced by electrical stimulation (synchronous release) or hypertonic solution (asynchronous release) occur at the active zone, in response to increasing depolarising concentrations of extracellular potassium they occur randomly dispersed on the membrane (Ceccarelli et al., 1988).

## 4 Lost in noise, or extracting information from noise?

Far back, Sir Katz and Miledi (1972) observed that the noise produced by acetylcholine (ACh) at the frog neuromuscular junction could be treated statistically by applying Campbell’s theorem (Rice, 1944), to extract the elementary event produced by the activation of a receptor molecule by ACh binding and estimate how many such events would contribute to the generation of a miniature end-plate potential (mEPP; about 5,000).

This approach was applied to several physiological questions and became a standard approach in estimating the conductance and kinetics of ion channels, before single channels recording through the patch clamp technique became standard practice (see, e.g., Conti and Wanke, 1975).

During my stay at the Rockefeller, we developed, together with Paul Hurlbut, John Segal and Bruno Ceccarelli, a procedure to apply a similar principle to recordings of quantal release at the neuromuscular junction, through an extension of Campbell theorem to higher semi-invariants (Segal et al., 1985). This way we obtained an estimate of the amplitude and rate of occurrence of mEPP during the asynchronous, high frequency quantal release produced by black widow spider venom (Fesce et al., 1986a); under this condition quantal release would often occur in bursts, so that specific theoretical and analytical approaches had to be developed to treat noise nonstationarity, to resolve the synaptic noise into its elementary components (Fesce et al., 1986b; Fesce, 1990; see also Heinemann and Conti, 1992). It seemed that the approach could be profitably applied to many other experimental questions and situations but, apart from a few studies in which it was used to measure junctional activity at the cyto-neural junction in the frog posterior semicircular canal (Rossi et al., 1994), the apparently promising procedure did not yield noteworthy fruits.

## 5 The freezing era: freeze-fracture and quick-freezing

**FIGURE 3:**
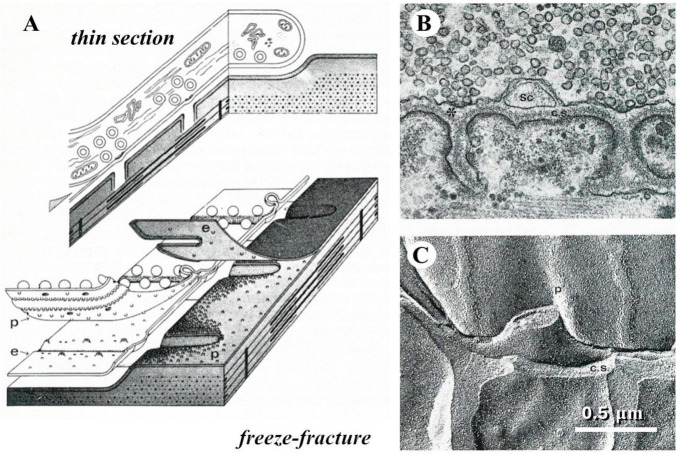
The freeze-fracture technique. **(A)** Diagrams that display how the plane of fracture tends to run in a frozen tissue; in particular, they illustrate the way a frog neuromuscular junction appears in thin section electron microscopy or after freeze-fracture. **(B)** Thin-section electron micrograph of a small portion of a frog neuromuscular junction. **(C)** freeze-fracture replica of a similar preparation, which illustrates the correspondence between the appearances of the same structures in the two kinds of electron micrographs.

When I started my career a novel and intriguing morphological technique had recently been introduced, referred to as freeze-fracture or freeze-etching (Park, 1972). The procedure took advantage of the different mechanical properties of frozen aqueous vs. lipidic components of biological tissues. Breaking with a razor blade a frozen preparation, the fracture plane tended to split membrane bilayers, thus following and exposing the surface of cells; these would be diagonally sprayed with platinum, so that a replica was created that appeared like a landscape covered by snow (Figure 3).

In our laboratory, this procedure turned out particularly useful in displaying the presynaptic membrane that became marked by dimples, indicating the fusion of synaptic vesicles, if the neuromuscular preparation was fixed while intense neurotransmitter release was being elicited. However, we would have liked to be able to capture the images of vesicle fusion in real time, and that was not possible using chemical fixation techniques.

This was made possible by taking advantage of the so-called rapid-freezing (quick-freezing) technique, based on slamming a preparation on a copper block held at extremely low temperatures with liquid helium. This approach was shown to preserve the cellular ultrastructure of a biological preparation to a depth of at least 10 μm from the impact surface. John Heuser developed a machine to deliver an electrical stimulus to the nerve during the descent of a neuromuscular preparation, mounted on a plunger, toward the copper block held at ∼4°K, so that the preparation was fixed at variable intervals (in the ms range) after the stimulus. Freeze fracture studies using this approach (Heuser et al., 1979) and thin sections obtained with the freeze-substitution procedure (Van Harreveld et al., 1965) on similarly treated preparations (Torri-Tarelli et al., 1985) were instrumental in demonstrating the temporal coincidence between synaptic vesicle fusion and spike induced neurotransmitter release.

This was another curious experience, because a lot of effort had gone in developing the instrument and the procedures and in making the experiments and analyzing the results, but once the coincidence of the electrophysiological and morphological events had been observed, little use remained for the machinery and the technique. As it had been the case for noise analysis of synaptic recordings, the innovation had become almost simultaneously promising and obsolete.

## 6 Here come the dyes: functional imaging using fluorescent probes

During the last 40 years innumerable functional dyes were developed and taught us a lot about physiological processes. The initial revolution were the calcium dyes, fluorescent compounds that would change their excitation or emission spectrum when bound to calcium ions. Quin2 came about in the eighties and was rapidly put to work in studying the relations between cytoplasmic Ca^2+^ and neurotransmitter release (Meldolesi et al., 1984). However, quin2 had quite low affinity for calcium, and the high concentrations needed would therefore buffer and blunt calcium transients. Higher affinity dyes were soon developed, such as fura-2 and indo-1 (Jackson et al., 1987). Systems to take the ratio between the fluorescence at two excitation (fura-2) or emission (for indo-1) wavelengths were developed to be able to measure calcium concentrations independently of the amount of dye loaded into the cells. Here, the story paralleled what was happening in many other fields: initially, several laboratories would develop mechanical devices to rapidly swap optical filters to alternate images taken with two excitation or emission wavelengths (e.g., Malgaroli et al., 1990); however this would not allow computing more than 1-2 ratios per second. Then came the monochromators, and tuneable-wavelength lasers, electronics relieving the mechanics of most of the work.

Calcium sensitive probes and proteins came as a revolution in the field of cell biology and neurobiology, especially thanks to the work of Tsien (1998), who engineered several mutants of the green fluorescent protein (GFP), from the jellyfish *Aequorea victoria*, so that it could be used as a calcium, redox, or pH sensor, or as a reporter for expression of specific proteins; this application flanked the firefly luciferase reporter gene, which has been widely applied in both transient and stable transfection protocols in eukaryotic cells to measure transcriptional regulation of DNA elements (Brasier and Ron, 1992). Specific mutations of the GFP protein also made it possible to change the emission spectrum, to obtain a turquoise form and a yellow one (YFP).

The idea that a fluorescent substance can change its light sensitivity, or emission, based on the local environment did actually give rise to innumerable applications: from sensors of each specific ion species, or of pH, to voltage sensitive dyes, lipophilic substances that migrate to cell membranes and change their fluorescence properties depending on membrane potential; or to studies of interactions, based on the capability of a substance to quench the fluorescence of another, or on the principle of FRET (Fluorescence Resonance Energy Transfer), where a fluorophore, which normally emits light at a certain wavelength, is quenched by, and excites, another fluorophore that will emit at a different wavelength, when the two are close enough (the mentioned turquoise and YFP can perform this trick).

## 7 Patch -clamp and its aftermaths

The procedure invented by Neher and Sakmann (1976), which earned them the Nobel prize for Medicine in 1991, was a true revolution in the field of neurobiology. Through the patch-clamp technique it became possible to obtain electrophysiological recordings without producing damage in the plasma-membrane, even in small neurons that would not tolerate the penetration of an electrode; more importantly, single channel recording in cell-attached or excised membrane patches made it possible to monitor the conformational modifications of a single protein in real time, for the first time. This essentially remained the sole technique capable of this for about three decades, until high-speed atomic force microscopy became practically usable to this purpose (Ando et al., 2007; Umeda et al., 2023).

The patch-clamp technique in its various forms – whole-cell, cell-attached, inside-out, outside-out recording – made it possible to gather an incredible amount of experimental evidence on voltage-gated as well as ligand gated channels, their gating kinetics, their conductance and their pharmacology. The properties of channels and transporters could be easily studied this way, at the single molecule level, by having them expressed in *Xenopus laevis* oocytes, a particularly versatile system, relatively poor in endogenous electrophysiologically active membrane proteins, and in the meantime an efficient expression system, which therefore also made it possible to perform mutations in channels and transporters, to clarify the contribution of specific residues to the binding, gating and conductance properties of the molecule of interest (Ivorra et al., 2022).

In addition to the unprecedented possibility to monitor and get to understand molecular dynamics, patch clamp technique opened a whole new field of study in cellular biology, thanks to the ingenuity of Erwin Neher, who realized that having a low-resistance access to the inside of a cell made it possible to determine the resistive and capacitive properties of the cell membrane, and consequently to measure its surface area (Neher and Marty, 1982). This way, secretory phenomena could be studied in quantitative terms, in real time.

Something more came out of this. In Alvarez de Toledo et al. (1993) were able, while visualizing the fusion of histamine containing granules, to measure the capacitance (and membrane surface) changes and simultaneously determine quantitatively the release of histamine through amperometry (measuring the redox current), in real time, with time resolution in the ms range. This way, they were able to demonstrate that release from a granule can occur even without it collapsing into the plasma membrane, through a reversible fusion of the granule and cellular membranes, and a transient opening of a pore between the vesicle lumen and the extracellular space. This first demonstration was obtained using beige mouse mast-cells, which possess giant granules, but other researchers reproduced similar studies in other cells (e.g., chromaffin cells) with smaller granules. Neher (1993) proposed an extrapolation of these data, obtained with granules of variable sizes, to synaptic vesicles, and concluded that a synaptic vesicle should be able to release most of its content through a transient, sub-millisecond, opening to the synaptic cleft. My mentor, Bruno Ceccarelli, who prematurely died in 1988, would have been very excited by these data, because he had heralded the possibility of quantal neurotransmitter release through a transient, reversible fusion of the synaptic vesicle – a process that we named “kiss-and-run” release (Fesce et al., 1994) – since his first reports of the capacity of synaptic vesicle to recycle (Ceccarelli et al., 1972, 1973).

In general, patch-clamp had profoundly changed the field of electrophysiology. It used to be the world of strange people who worked in the dark, their head hidden in Faraday chambers, in perennial fight against 50-Hz (or 60-Hz) noise, masking and grounding everything, afraid that people would come in the room and touch something or simply talk too loudly, so that the electrode would imperceptibly move and lose the impalement or destroy the cell. Now, it seemed that everybody could do electrophysiology, with this new technique. And gradually came the most sophisticated piezo-controlled micromanipulators and visualization improvement, so that machines would do all the work, positioning, repositioning, contacting the membrane, and you only had to apply the appropriate suction to establish the “giga-seal” or perforate the cell membrane. Electrophysiology was gradually losing its aura of magic and mystery, when a new revolution came, the multielectrode arrays (MEA) mounted in the floor of modified Petri dishes. It was now possible to simultaneously record the activity of many neurons (or cardiomyocytes) in culture, or in a brain slice, and anybody could do it, on a simple table, in the middle of a crowded room. But if electrophysiology had become much less demanding, it certainly was even more fruitful.

More recently, the discovery in bacteria, and transfection in the cell of interest, of photosensitive channels, and more in general proteins that could change conformation in response to light (“photosensitive switches”), has generated the revolution of “optogenetics,” with the possibility to activate – or shut off – cellular processes at will and with precise timing, through a laser beam (Armbruster et al., 2024). Photosensitive channels, in particular, can be transfected in specific neurons. This has added a new dimension to electrophysiological studies, making it possible to investigate the effect of the activation of specific neurons or circuits – also *in vivo* – and their involvement in particular information processing steps.

## 8 … and IT, computers, networks, AI

Possibly the most astonishing innovation that accompanied all my research life was the impressive, continuous evolution of computers. One used to need scissors and scotch tape to modify a manuscript, actual “cutting” and “pasting,” not simply clicking a couple of keys; and retyping, and typing again. I loved my cupboard-sized up-to-date PDP computer, though it used to take 2 min to perform a 4096-points fast (!) Fourier transform, and we, the “scientists,” had the privilege of being able to communicate with each-other, overseas, taking advantage of the ARPANET, the precursor of internet and the world-wide web. Then, every year there would be a new processor, a new storage system (can you imagine the 512 kB 8-inch floppy diskettes, now that you have a 1-TB ½-inch usb key in your pocket?), a new operating system (which however keeps taking 2 min to turn on, and crashes or decides it wants to update in the middle of your lecture or presentation). So, a geometrical increase in speed, performance, data capacity. But the most fascinating evolution in the field is possibly the idea of dynamic programming, i.e., writing software capable to modify itself – in a guided manner or even blindly – to manage to exactly produce the wanted result, or correctly classify or predict. This – changing processing mode based on previous activity, as our neurons do – is the principle of machine learning, which is making computers even more proficient than human beings in investigating, collecting data, classifying, interpreting and predicting.

It is obvious that there are differences between the way a machine learns and the way we do, and we think, although artificial intelligence (AI) systems such as ChatGPT let us think that the machine capable of passing the “Touring test” (tricking a human into thinking that it is also human) has eventually come.

In the future of AI, two quite different paths can be seen. On the one hand, AI systems still miss a sufficiently sophisticated emulation of human emotions, and somebody is trying to fill this gap, essentially pursuing a proof of principle, like alchemists in search for philosopher’s stone; and this may raise quite hard questions about rights, diversity, dignity and justice, if we manage to build the “human” machine, capable of suffering. On the other hand, the capacity to handle huge sets of data and apply impeccable logic makes computers quite superior to humans, who can only analyze no more than 7–9 items at a time in their reciprocal relations, and who disagree on most interpretations and decisions – even the merely technical and scientific ones. But this is because all human interpretations and decisions arise from a process of continuous re-evaluation of the available data under different perspectives. The brain takes into consideration a limited number of aspects at a time and the relevance of each aspect changes at any moment, as a function of the other factors that are being considered and the emotional state that the evaluation process is producing at every moment; thus, our interpretations and decisions are tentative, based on likelihood, amendable. In a mathematical world artificial intelligence would certainly be much better than the human one, but the world we live in – though it certainly seems to obey the laws of physics – is full of the variability, unexpectedness, uncertainty and surprise that biology has accustomed us to, and doubting, not being so certain, may in the end be something good.

Still, machine learning and AI, on one side, and the spectacular developments in brain imaging, especially functional imaging, have transformed cognitive neurosciences into a whole new extraordinarily prolific field and certainly have changed our perspective on the possibility of understanding how the brain works. Cellular neuroscience, on the other side, though the large public has been much less aware of the incredible amount of information the scientific community has acquired on the cellular aspects of neuronal function and neural circuit activity, may indeed help us to eventually understand the mechanistic detail of how the brain work.

But this is no more in the past, this is the future.
